# Spirituality in a Doctor’s Practice: What Are the Issues?

**DOI:** 10.3390/jcm10235612

**Published:** 2021-11-29

**Authors:** Ángela del Carmen López-Tarrida, Rocío de Diego-Cordero, Joaquin Salvador Lima-Rodríguez

**Affiliations:** 1 Department of Critical Care and Emergency, Hospital Saint John of God Aljarafe, 41930 Seville, Spain; angelacarmen.lopez@sjd.es; 2 Research Group CTS 969 Innovation in HealthCare and Social Determinants of Health, Faculty of Nursing, Physiotherapy and Podiatry, University of Seville, 41009 Seville, Spain; joaquinlima@us.es

**Keywords:** spirituality, religiosity, spiritual care, physician, doctors, health professionals, comprehensive care, holistic

## Abstract

Introduction: It is becoming increasingly important to address the spiritual dimension in the integral care of the people in order to adequately assist them in the processes of their illness and healing. Considering the spiritual dimension has an ethical basis because it attends to the values and spiritual needs of the person in clinical decision-making, as well as helping them cope with their illness. Doctors, although sensitive to this fact, approach spiritual care in clinical practice with little rigour due to certain facts, factors, and boundaries that are assessed in this review. Objective: To find out how doctors approach the spiritual dimension, describing its characteristics, the factors that influence it, and the limitations they encounter. Methodology: We conducted a review of the scientific literature to date in the PubMed, Scopus, and CINAHL databases of randomised and non-randomised controlled trials, observational studies, and qualitative studies written in Spanish, English, and Portuguese on the spiritual approach adopted by doctors in clinical practice. This review consisted of several phases: (i) the exclusion of duplicate records; (ii) the reading of titles and abstracts; (iii) the assessment of full articles and their methodological quality using the guidelines of the international Equator Network. Results: A total of 1414 publications were identified in the search, 373 of which were excluded for being off-topic or repeated in databases. Of the remaining 1041, 962 were excluded because they did not meet the inclusion criteria. After initial screening, 79 articles were selected, from which 17 were collected after reading the full text. A total of 8 studies were eligible for inclusion. There were three qualitative studies and five cross-sectional observational studies with sufficient methodological quality. The results showed the perspectives and principal characteristics identified by doctors in their approach to the spiritual dimension, with lack of training, a lack of time, and fear in addressing this dimension in the clinic the main findings. Conclusions: Although more and more scientific research is demonstrating the benefits of spiritual care in clinical practice and physicians are aware of it, efforts are needed to achieve true holistic care in which specific training in spiritual care plays a key role.

## 1. Introduction

Addressing a person’s spiritual dimension in clinical practice during illness, healing, and the end of life brings the patient an added benefit that positively links spiritual care and physical and mental well-being, as people with more developed feelings of spirituality more resiliently cope with the challenges they face [1,2,3,4]. 

Previous studies have shown that patients show a positive interest in having their doctor address questions about their spiritual needs [5,6,7] as they find that it strengthens their relationship; helps the doctor better understand the person by knowing their values, convictions, and attitudes; and aids consensual decision-making [8,9].

Attention to the spiritual dimension in clinical practice centres around two fundamental ideas: firstly, the specific definition of what spirituality is, and second, why the doctor should care for it in his or her care-giving relationship with the patient. Regarding the definition of spirituality, the main confusion lies in the concept of religiosity; while spirituality could be defined as the search for meaning and purpose in life, self-transcendence, and connections with others and the world around us, religiosity could be defined as the set of beliefs and practices of an organized religious institution, as well as membership of and participation in different organized activities such as rituals and other activities connected to a particular religious faith [10]. These terms are not mutually exclusive. Rather, they overlap and even coexist, which is where the confusion between the definitions seems to lie. The spiritual dimension should be addressed in healthcare because consensual decision-making between doctor and patient is one of the cornerstones of the current healthcare model. Knowledge of a patient’s values and needs from the spiritual dimension favours the adequate provision of holistic care and has become, in practice, an ethical duty [11,12].

There have been an increasing number of studies on the role of spirituality in medicine. Spirituality is understood as a vital human need that must be valued and taken seriously, especially in situations of adversity such as illness in which the person questions the very meaning of their existence, transcendence, or suffering [13,14,15]. It is therefore essential to address these spiritual needs in an integrated way with a care giving model in which all facets are considered and supported by a multidisciplinary team [16].

Although there are quantifiable and demonstrable data on the health benefits of spiritual care giving, there are still many professionals who tend to avoid this topic in clinical care, even though most patients would like to incorporate the spiritual dimension more in discussions with their doctors [1,7,17,18].

Considering all of the above, the main objective of this study was to find out how doctors approach the spiritual dimension by describing its characteristics, the factors that influence it, and the limitations they encounter.

## 2. Materials and Methods

### 2.1. Protocol

Our work, conducted while following the PRISMA guidelines, is a narrative synthesis of the studies that evaluate the perspective of doctors regarding spirituality in the care giving they administer in their clinical practice. The protocol was previously registered in PROSPERO under registration number: CRD42021240704.

### 2.2. Databases and Search Strategy

Studies were searched in the following international electronic databases: PubMed, Scopus, and CINAHL. During the study period (from February to April 2021), the same search strategy was used in all consulted data bases. This strategy was based on the DeCS/MeSH descriptors of the Health Sciences that located, selected, and compiled the results and findings of the original studies within the context of the study’s objective (Table 1).

Articles were included if they were peer-reviewed articles: (a) with original data; (b) with full text access; (c) were published in Spanish, English, or Portuguese; and (d) that met the PICOTS criteria (Table 2). Articles were excluded if they were: (a) duplicated in other databases; (b) notes to the editor, reviews, instrument validation, book chapters, clinical cases, narrations, dissertations, opinion pieces, or not found; or (c)of a low methodological quality after controlling for biases. Studies exclusively dealing with medical interns (resident doctors) were also excluded because we decided they did not have sufficient experience in medical practice.

### 2.3. Study Selection and Data Extraction

After the search, all the references were included in the Mendeley bibliographic reference manager (version 1.19.8, University of Seville, Seville, Spain). Initial screening was conducted by excluding all duplicated publications and then reading the titles and abstracts. The screening procedure was independently carried out by two reviewers in order to identify potentially relevant studies based on the inclusion and exclusion criteria. Where there was a lack of consensus regarding the quality of an article, a third reviewer was consulted.

After this first stage, the articles to be included were submitted to a full-text reading by two reviewers independently. The articles included studies that analysed the approach to spiritual care in clinical practice by physicians, as well as the barriers or limitations encountered by physicians in the spiritual dimension of care giving. When doubts arose about any of the described procedures, the authors of the articles were contacted by email.

Finally, the following items were extracted from each study: authors, year, country, sample characteristics, design, purpose, and main results. The resulting tables were independently and thoroughly checked by three reviewers, who engaged in critical discussions about the data.

### 2.4. Quality Assessment

Although not required, the guidelines of the international Equator Network were considered to assess the methodological quality of the selected studies. The Consolidated Criteria for Reporting Qualitative Research (COREQ) were used to assess the reporting of interviews and focus groups [19] and the Strengthening the Reporting of Observational Studies in Epidemiology (STROBE) was used to assess the reporting of observational studies (cross-sectional studies) [20].

The guidelines of the international Equator Network do not specify a minimum score for determining the methodological validity of a study. However, in this review, the methodological quality of studies was considered to meet a medium-high value if STROBE scores reached a mean of 13/22 points for descriptive observational studies and if COREQ scores reached a mean of 19/32 points for qualitative studies.

The included studies were independently assessed by two reviewers for methodological validity prior to inclusion in the review. Any disagreements that arose between the reviewers were resolved through discussion or by a third reviewer.

**Figure 1:**
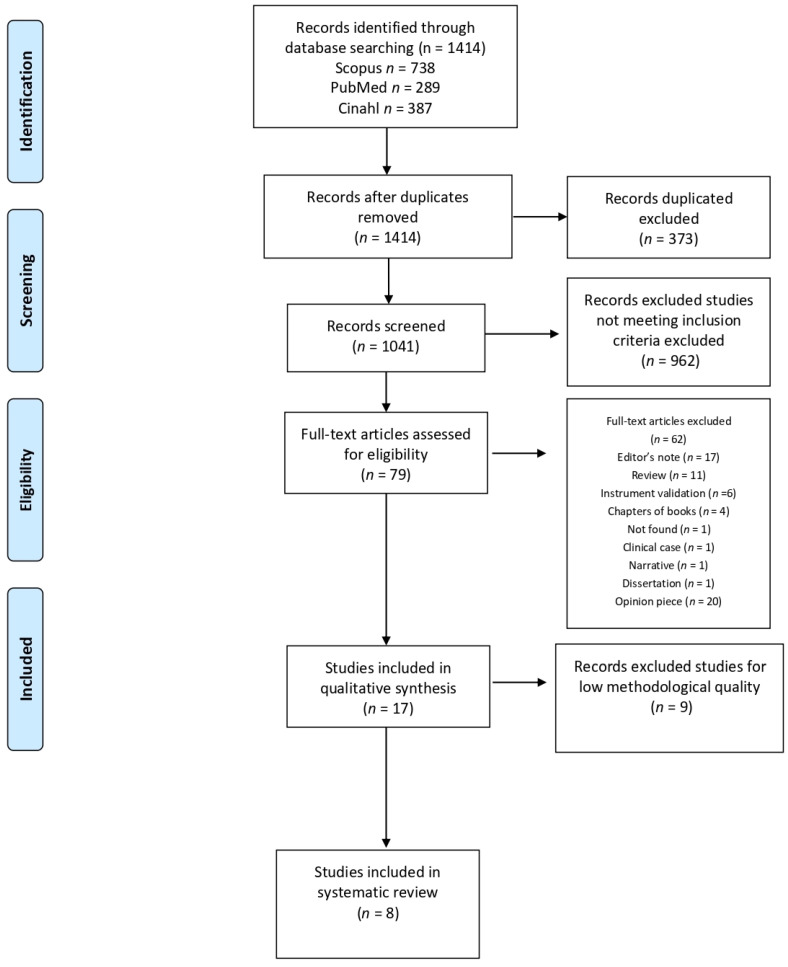
Selection process flowchart.

In the three databases (PubMed, Scopus, and CINHAL), the search process identified 1414 potentially relevant publications matching the eligibility criteria (PICOTS). After removing duplicates, 1041 articles remained, of which a further 962 articles were excluded after screening the titles and abstracts. Next, a total of 79 articles underwent full-text analysis. After reading all the records from the two independent reviewers, 62 were excluded because they were editor’s notes (17), reviews (11), instrument validations (6), chapters of books (4), clinical cases (1), narratives (1), dissertations (1), opinion pieces (20), or not found (1). Nine articles were also excluded due the poor standard of methodology used. Finally, eight articles were selected (Figure 1).

## 3. Results

### 3.1. Descriptive Characteristics of the Studies

We included eight articles that explored the physicians’ perspective of the spiritual dimension of care giving in clinical practice, three of which were qualitative studies and five of which were observational descriptive studies.

A total of four of the articles had been published in the last six years (between 2017 and 2021), and four of all selected studies were multicentre studies.

The articles were from the USA (2), European countries (2), Canada (2), Asian countries (1), and Australia (1).

In relation to the sample size, there was a wide variety in the number of subjects, ranging from a minimum size of 6 [21] to maximum size of 1156 [22].

It was further appreciated that most of the studies were carried out in the area of palliative care (4). The areas of the remaining studies were oncology (1), primary care medicine (1), and various medical areas such as internal medicine, surgery, gynaecology, and paediatrics. In relation to the gender of the participants, only three of the studies had a majority of female samples, while in relation to the religiosity of the surveyed professionals, the clinicians in all studies considered themselves to be very or moderately religious.

Accordingly, the results showed which instruments (such as scales and surveys) were used to assess how spirituality is addressed in clinical care, and it was observed that three of the cross-sectional studies (37.5%) used validated scales to explore the practitioner’s attention to the spiritual dimension—two of them having been published in the last five years (2017–2021). The scales used to assess practitioner spirituality were: the Religious and Spiritual Beliefs and Practices Scale developed in 1999 by Daaleman and Frey [23], as part of an ad hoc survey [24], the Spirituality and Spiritual Care Rating Scale (SSCRS, devised by McSherry et al., 2002 [11,25], and the Duke University Religion Index (DUREL) developed by Koenig and Bussing [26] together with Suzuki and Kino’s Multidimensional Empathy Scale (MES) [27,28] (Table 3).

### 3.2. Synthesis of Results

The five selected descriptive studies analysed the beliefs, attitudes, and behaviours of physicians in addressing the spiritual dimension. Three studies evaluated physicians with different religious beliefs, the majority being Christian (Catholic or Protestant), in oncology in Australia [25], different clinical areas in the USA [22], and palliative care in the Netherlands [24]. Hamouda et al. [27] analysed the spiritual care of Muslim physicians in palliative care in the USA, and Al Yousefi [29] analysed the spiritual care of Muslim physicians in different clinical areas in Saudi Arabia. The samples were mostly represented by male participants, and the mean age was 45 years.

Among the main findings, it was found that more spiritual physicians were more sensitive and performed more appropriate spiritual care. In fact, the study by Al Yousefi [29] found that the majority of respondents (91.1%) thought that the influence of religion on health was generally positive. Only in one of the studies was this not the case [27], as there was no positive correlation between physicians’ spiritualistic beliefs and better spiritual care (in contrast to the other study that was conducted with Muslim physicians [29]. One study [22] found that physicians’ willingness to attend to patients’ spiritual needs varied according to the religious concordance between clinician and patient; if both practiced the same religion, they were more likely to discuss these issues in the clinical relationship. This study demonstrated that patients want to discuss these issues with their clinicians, and clinicians reported that attending to the spiritual dimension of their patients improved the trusting relationship in clinical practice.

Clinicians consider spiritual care a good practice, although in two of the analysed descriptive studies, more than 50% of respondents reported that they did not address this [24,29]. In another [14], 45% of clinicians considered themselves capable of addressing it themselves, although 90% chose to refer to expert providers such as chaplains, counsellors, or pastoral agents. This same study analysed confusion in addressing spirituality in a clinic through the lens of the physician’s competence, which generated indecision and made it difficult to manage in practice and refer to others. The authors of the study also suggested the use of brief tools to address the spiritual needs of patients in the absence of time to address their spiritual needs.

On the other hand, insufficient time and a lack of training were identified as the main barriers for physicians to address the spiritual dimension [22,24,25,27,29] although the lack of a suitable environment [29], fear of offending the patient they care for [27], and not considering it their responsibility [24] were also highlighted.

In the three selected qualitative studies based on semi-structured interviews, the samples were mostly women and neither religious affiliation nor age was specified, although the time in clinical practice was specified with a mean of 15 years.

One study on primary care medicine in the United Kingdom found that physicians reported that they did not usually address these types of issues in their routine practice and alluded to a lack of professional and personal experience in this regard or that they did not believe it was within their clinical competencies [30]. Penderell and Brazil’s [21] study of palliative care physicians in Canada discussed the concept of spirituality and how physicians view it in their practice as a positive factor for the health of their patients and themselves from the professional and personal points of view. In the other study also conducted on Canada in palliative care [32], physicians were found to value spiritual care as fundamental to alleviating suffering and promoting healing in their patients, thus nurturing personal spirituality.

## 4. Discussion

The main objective of this study was to find out how doctors approach the spiritual dimension by identifying its characteristics, the factors that influence it, and the limitations doctors encounter in this dimension of care giving.

The results showed that the vast majority of doctors are positively disposed towards incorporating the spiritual dimension into patient care (although in two study, the doctors were less involved) and recognize its direct effects on improving the quality of life and medical care. The main difficulty in approaching the spiritual dimension among doctors was reported that if it is to be addressed in clinical practice, it is necessary to clearly define what spirituality is, why it is relevant in health care, and how it is to be valued.

A significant proportion of physicians reported believing that spirituality belongs to the intimate sphere of a person and failing to consider that it plays a key role in attitudes and decision-making when an individual is faced with serious illness (as well as other facets of their lives). While it is true that spirituality forms a part of the intimate sphere of the individual, it is also a universal value that is present in all people and affects them when they are ill just as importantly as the physical, psychological, and social spheres, which is why it must also be addressed, as shown by other studies [33,34]. Patients ask their doctors to address their spiritual needs because they need to feel that someone has listened to them and cares about their problems, worries, and concerns when they are faced with the challenge of a serious illness or even the end of their life [35,36]. This attention strengthens the patient–doctor relationship and, from an ethical point of view, favours joint clinical decision-making, as well as respecting the patient’s autonomy and dignity and ensuring that the care given in the clinical practice is both holistic and humanized [11,12]. This was also reported in two of the analysed studies [21,32].

Given the complexity of giving a suitable definition of spirituality in clinical care, no homogeneous way of assessing it was found in the reviewed articles [25], addressed this same issue and considered it a barrier that causes a physician to not to attend to spiritual needs and/or to refer their patient to expert providers (such as chaplains, counselors, and pastoral agents).Some studies discussed the wide terminological diversity, and often out right confusion, over the concept of “spirituality” in healthcare [37,38,39,40,41,42,43]. This difficulty seemed to translate into the way the concept has been analysed since some of these viewed descriptive studies used surveys and questionnaires designed for the purpose of the study (ad hoc), while others used validated scales that offer interesting objective data without going deeper and without providing consensus or uniformity over what the standard scale for assessing spirituality should be. In this respect, the methodology of qualitative studies that used semi-structured interviews and content analysis allowed for more in-depth and detailed information to be obtained on the research topic. There is evidence that for the analysis of complex research questions such as the case at hand, a combination of quantitative and qualitative perspectives allows for the greater depth and understanding of the topic of study and confers greater consistency and scientific rigor [44,45,46].

From another point of view, those who consider spirituality to be a private part of an individual’s character may be hesitant to address it because their own spirituality is not particularly developed. It has been shown that physicians with a more developed spirituality provide their patients with better care in this dimension [8,47,48,49,50,51] and give the patient more opportunities for dialogue and being listening to in a symmetrical and reciprocal person-to-person relationship than the relational asymmetry that often occurs. Such opportunities for dialogue should form the basis of future training in this area. It was noted that spiritual care is closely related to the physician’s own spirituality and shown that a physician with a greater sense of spirituality/religiosity is more likely to more adequately attend to their patients’ spiritual needs [22,29].In contrast, the study by Hamouda et al. [27] conducted on Muslim physicians showed that there is no direct relationship between physician spirituality/religiosity and better spiritual care, and the authors associated it to a socio cultural factor in this community. In contrast, the study by Al Yousefi [29], also conducted on Muslim physicians, showed the opposite, so it seems that this reasoning is not the most accurate. In this regard, there were other studies that showed that when faced with existential questions from their patients, the responses of doctors ranged avoidance, fear of harm, and overprotection to sometimes inadequate management due to a lack of skills, all of which generate distress, bewilderment, or even pain in patients [6,52], hence the extreme importance of adequate training as advocated by some of the analysed studies [25,27], as in previous [14,53,54,55,56]. In addition, in most of these viewed studies, physicians were found to be less active in addressing these types of patients’ needs, not only because of the barriers described above but also because they were mainly trained in scientific–technical skills for the traditional biomedical model focused on objective, measurable data.

Regarding the characteristics of physicians that influence spiritual care, apart from their spiritual/religious beliefs, none of the studies analysed differences in relation to gender or age, for example. It seems that it is women and physicians with more clinical experience who are more inclined to provide spiritual care. We believe that due to their anthropological characteristics and social and cultural commitment, women are more prone to engage with these types of issues. We think that the sum of a physician’s clinical experience and own life experience may make them more inclined to consider transcendence and the meaning of life, which makes them more openly discuss these issues with patients.

In addition to considering that spirituality is an intimate part of an individual’s personality that has nothing to do with the clinical relationship, doctors also commented on the lack of time in which to attend to the patients with respect to this dimension [57,58,59,60] due to the standardized protocols that do not allow it, with patients often being referred to other professionals who have more experience in this area. Since greater importance is attached to the other aspects of their clinical work within the healthcare practice, doctors tend to placeless importance on spiritual matters or avoid them altogether, delegating them to others (such as chaplains, counsellors, and pastoral workers) and neither taking on responsibility nor engaging in management training, as stated in other consulted sources [61,62]. A study by Kichenadasse et al. [25] suggested possible solutions to the expressed difficulty of a lack of time, proposing the use of brief spiritual assessment tools in patients such as the Faith/Beliefs, Importance, Community, Address in care or action (FICA); Hope, Organised religion, Personal spirituality, Effects of care and decisions (HOPE); and Spiritual belief system, Personal Spirituality, Integration, Rituals/restrictions, Implications, and Terminal events (SPIRIT), which could facilitate assistance in both the detection of spiritual needs and the referral to spiritual care practitioners in an expedited manner. This proposal seems to favour an initial evaluation of a person’s spirituality complemented by adequate training in the care of their spiritual needs.

Similarly, in a study observing health science students, it was found that the main barriers encountered in practice with their patients were time limitations, fear of offending patients, and a lack of specific training during their academic training to address the spiritual dimension [39]. These barriers were also identified in other articles that specifically analysed the difficulties encountered by health professionals (such as physicians, nurses, social workers, and psychologists) in addressing the spiritual dimension, and areas were proposed for improvement [57,58,59,60,63,64].

On the other hand, nurses were reported to cater to the concepts of spirituality and religiosity while caring for their patients from the beginning of their professional training, which gives them greater sensitivity when addressing these needs. Based on key theories such as Jean Watson’s Transpersonal Care Theory (which encourages nurses to go beyond the procedures, tasks, and techniques used in their daily practice), nurses understand that spirituality is part of their integral care. Transpersonal Care Theory combines sciences with humanities and cross-cultural understanding in a mind–body–spiritual framework, with a phenomenological, existential, and spiritual orientation [65,66,67]. For other authors of studies on nursing such as Sawatzky and Pesut, spiritual expressions such as love, hope, and compassion constitute the most basic universal approach to spiritual care and can be integrated into all aspects of nursing care [10].

The clinical tools used in nursing, such as the NANDA taxonomic classification (2020) that recognizes the Nursing Diagnosis of “Spiritual Distress” (00066) or the Diagnosis “Risk of Spiritual Distress” (00067), the NOC classification of outcomes that includes the outcome “Spiritual Health” (2001), and the NIC classification of interventions that includes the intervention “Spiritual Support” (5420), are all examples of this integration of the spiritual dimension into clinical practice. When consulting other references, we found comparisons in spiritual health care dynamics between physicians and nurses, resulting in nurses being more sensitive to spiritual care than physicians [57,68,69,70,71,72,73], perhaps due to this fact regarding their academic training.

In this regard, Sajia and Puchalski [74], for instance, advocated the development of educational competencies for the spiritual dimension of healthcare and proposed guidelines for developing this skill as part of a physician’s curriculum in addition to the other scientific–technical skills required for their normal clinical practice. On the other hand, Koenig [31,36,75] advocated for improving the quality of clinical care by incorporating spiritual care into health systems, as well as assessing and offering solutions for the possible barriers encountered by medical teams when considering this dimension in practice.

Although some of these viewed studies focused on the perspective of physicians in their spiritual care in clinical practice, this point of view was contrasted with that of other health professionals’ other studies. In two recent studies, we found the explicit defence of a spiritual approach from multidisciplinary care to be a priority in the care of these patients [76], and physicians have even described this approach as a facilitating factor in the support of the multidisciplinary team for adequate spiritual care [64]. Similarly, we believe that in order to promote the spiritual health of patients to a level that suitable and adequate, the multidisciplinary approach (in which one assesses the patient from different perspectives, including the spiritual aspect) is fundamental and ethical.

Finally, most of the publications focused on the healthcare areas of palliative care, oncology, and intensive care units [2,77], where the need for spiritual care could be said to be more urgent, though others did not clearly specify which health sector the study had been conducted in. However, spirituality is not exclusively limited to these areas of healthcare, since all patients tend to ask a wide range of questions to help them come to terms with the experience of being ill and doctors have a professional duty to respond to these needs. This idea is supported by examples in other studies that assessed the importance of addressing the spiritual needs of patients who are treated in other medical specialties [78,79,80,81]. 

## 5. Practical Implications

To detect and resolve these difficulties in practice, we would like to put forward the following arguments and strategies as a basis for reflection and action:

### 5.1. The Notion That ‘The Patient’s Spirituality Is None of Our Business”

This notion is likely to be rooted in a confusion between the concepts of spirituality and religiosity. Every person is spiritual by nature, but not every spiritual person is religious. If the spiritual dimension of a person manifests itself through a transcendent relationship with God (through a creed and dogma), then it is a religious dimension. Indeed, spirituality is an intimate and innately human quality belonging to each person; it is a deeply-felt inner desire to forge a connection with everything that surrounds us because we need to find a meaning in our existence and the world in which we live. In situations of pain and suffering, when people are facing severe illness and death, this aspect is just as important as physical, psychological, and social aspects. Of course, it also needs to be addressed with great care.

### 5.2. Time Limitations

Addressing a patient’s spiritual needs is not given the importance it deserves by the established clinical protocols of action, which prioritize objectifiable scientific data; for this reason, extra time and physical space need to be found in order to meet these needs.

### 5.3. Referral to Other Providers with Spiritual Expertise

Health professionals often delegate the responsibility for spiritual care to chaplains and counsellors who are assumed to be more skilled in this area [82]. However, it is a physician’s ethical duty to consider a patient’s autonomy in clinical decision-making and a knowledge of their convictions, values, and spiritual needs makes for a stronger and more effective clinical relationship.

### 5.4. Lack of Specific Training

Suitable training that provides tools for the spiritual care of both practitioner and patient seems to be of paramount importance [43,56,83]. Training in spiritual care should be included in the curriculum, starting with the initial academic training of doctors right up to their final specialization.

Research strategies should be directed towards doctors or health teams containing doctors with the aim of developing long-term training plans in these competencies and the subsequent analysis of the results. When collecting data, it would be interesting to use mixed methods to obtain more in-depth information in order to lend greater consistency and scientific rigor to the study.

In addition, for more thorough analyses, it would be beneficial to assess the perspective of patients being cared for by doctors who have undergone this specific training in spiritual care and to observe the short-to-medium-term outcomes.

## 6. Limitations

The present review has certain limitations that must be acknowledged. Firstly, only three international databases were used to analyse the study aims, which made it impossible to compile a large number of records. Second, the search strategy used for this analysis narrowed down the search and excluded other synonymous terms. Third, in relation to language, studies not written in the languages of the inclusion criteria were omitted despite their possible relevance, and they were therefore not used in the analysis. Fourth, we sometimes did not have access to the full text. Finally, there was a certain degree of confusion in the terms used by the authors when indexing studies, especially between ‘spirituality’ and ‘religion’ and between ‘healing’ and ‘care’.

## 7. Conclusions

There are certain differences in the perspectives, beliefs, and practices of doctors and other health professionals (e.g., nurses), and although the general position of doctors is in favour of incorporating the spiritual dimension into health care, few actually do so at present. However, most studies have focused on the obstacles encountered by health professionals (such as doctors and nurses) in dealing with spiritual care, and few were found to contain proposals to remedy them.

Our conclusions are:

1. Doctors are aware of the fact that patient care involves many dimensions, including spirituality, but they encounter obstacles in their care practice to provide adequate care in this regard.

2. One of the main obstacles reported by clinicians is a lack of time and space to explore this delicate subject in greater depth, as it is not included in the standardized, routine clinical protocols of healthcare practice. Although there is a growing awareness and sensitivity towards spiritual care, as has been demonstrated in the recent scientific literature, it is not given sufficient consideration in global health care plans. Greater awareness and interest on the part of health managers and professionals in incorporating spirituality into clinical practice would result in spirituality playing a more relevant role in integral health care.

3. Some doctors consider that spirituality is part of an individual’s private life and therefore should not be addressed in health care. This causes them to be unwilling to intervene or even to refer patients to expert providers (e.g., chaplains) if the patients request it. In this respect, it should be noted that those physicians who consider themselves to be more spiritual/religious and whose own spirituality is more developed are more likely to be more responsive to these needs.

4. The main and most important barrier reported by the doctors is specific training in providing spiritual care, which in the whole cycle from initial academic training to specialization is not seen as an integral part of the doctor’s knowledge. The different studies highlight the lack of adequate training in spiritual care and the need to improve this aspect in order to include the spiritual dimension in daily clinical practice. This training is essential for offering high-quality, comprehensive patients care, although it has not yet been sufficiently incorporated into professional education. Scientific and technical skills are an essential requirement for a doctor, and competencies in other more patient-centred aspects, such as spiritual care, are also essential for a quality professional education to keep up with the times and meet the current needs of patients.

5. Due to the difficulty of defining it, multiple instruments have been designed to adequately assess approaches to spirituality in clinical practice [11,15,23,26,28,77,84,85,86,87]. We believe that for a good attention to the spiritual dimension, it is necessary to make a proper assessment. There are a wide variety of validated scales on how to measure spirituality, both in patients and among professionals, that have demonstrated the scientific possibility of evaluating this complex quality with objective, measurable data. These scales useful tools for addressing the use of spirituality in healthcare and estimating the spiritual needs of patients. Their standardized use would facilitate this dimension of health care, and their incorporation into clinical protocols would help to provide more comprehensive care for patients.

6. Several of the selected studies compared the perspectives of physicians with those of other health professionals. These studies showed that while physicians understand the importance of spirituality in clinical care, others, other such as nurses integrate it more into practice. A multidisciplinary approach favours an environment enriched by different perspectives on care giving and different attitudes towards spirituality in this context. In this way, truly comprehensive care is ensured when all a person’s facets are taken into account, including the physical, psychological, social, and spiritual dimensions. Coordination and joint work in spirituality would ensure the quality and continuity of such care.

7. We found no randomized clinical trial studies that specifically explored intervention strategies for physicians in spiritual care (either for themselves or their patients) and the outcomes obtained after their implementation. We believe that studies with this aim could promote a more scientifically rigorous approach to these outcomes, thus opening up new avenues of research that could explore spiritual care in physician-led clinical practice in greater depth and impart the scientific relevance and importance it truly deserves.

## Figures and Tables

**Table 1:** Strategy search.

Database	Query	Search Details
PubMed	physician AND (“spiritual care” OR “spiritual healing” OR “spiritual therapies”)	((((((“physician s”[All Fields] OR “physicians”[MeSH Terms] OR “physicians”[All Fields] OR “physician”[All Fields] OR “physicians s”[All Fields]) AND ((“spiritual”[All Fields] OR “spiritualism”[MeSH Terms] OR “spiritualism”[All Fields] OR “spirituality”[MeSH Terms] OR “spirituality”[All Fields] OR “spiritualities”[All Fields] OR “spirituality s”[All Fields] OR “spiritually”[All Fields] OR “spirituals”[All Fields]) AND “care”[All Fields])) AND “OR”[All Fields]) AND (“spiritual therapies”[MeSH Terms] OR (“spiritual”[All Fields] AND “therapies”[All Fields]) OR “spiritual therapies”[All Fields] OR (“spiritual”[All Fields] AND “healing”[All Fields]) OR “spiritual healing”[All Fields])) AND “OR”[All Fields]) AND (“spiritual therapies”[MeSH Terms] OR (“spiritual”[All Fields] AND “therapies”[All Fields]) OR “spiritual therapies”[All Fields])) AND (review[Filter])
Scopus	physician AND (“spiritual care” OR “spiritual healing” OR “spiritual therapies”)	TITLE-ABS-KEY (physician AND (“spiritual care” OR “spiritual healing” OR “spiritual therapies”))
CINAHL	physician AND (“spiritual care” OR “spiritual healing” OR “spiritual therapies”)	TITLE-ABS-KEY (physician AND (“spiritual care” OR “spiritual healing” OR “spiritual therapies”))

**Table 2:** PICOTS (population, intervention/exposure, comparator, outcome, time, and study design) criteria.

	PICOTS Criteria
Population	Physicians
Intervention/Exposure	Spiritual interventions
Comparator	No intervention/Waiting list/Usual practice/Placebo
Outcome	Health outcomes in physicians
Time	Without restrictions
Study design	Randomized controlled trials/ No randomized controlled trials/Observational studies/ Qualitative studies

**Table 3:** Main results found.

Authors, Year, Country	Design and Sample	Aim	Scale /Instrument	Main Findings
Al-Yousefi, 2012, Saudi Arabia [29]	Cross-sectional descriptive multicentre study *n* = 225 (physicians)	To assess the beliefs and behaviours of Muslim physicians regarding religious discussions in clinical practice and to understand the factors that facilitate or impede the discussion of religion in clinical settings.	Ad hoc survey	First study of this type carried out in a Muslim population. Most think that religion (not spirituality) has a positive influence on health, and more than half donot ask about this aspect in clinical practice. Doctors with a more careful religiosity approached it more easily; this coincided with those of greater age and experience.Spiritual care is mainly not approached due to lack of training and ethical dilemmas. Other barriers highlighted include: insufficient time and unsuitable environment.
Cocksedgeand May, 2009, UK [30]	Qualitative study *n* = 23 (physicians)	To explore the limitations of spiritual care in primary care physicians through two concepts: touch and spiritual care.	Semi-structured interviews	They identified barriers such as a lack of awareness of spiritual care, a lack of training, and thinking that other interests are more important to the person they serve.
Gijsberts et al., 2020, Netherlands [24]	Descriptive cross-sectional study *n* = 284 (physicians)	To examine the perceptions and experiences related to the provision of spiritual care at the end of life of elderly care physicians in nursing homes in the Netherlands, as well as factors associated with the provision of spiritual care at the end of life.	Ad hoc survey and Religious and Spirit Beliefs and Practices Scale (RSBPS)	Most perceived spirituality as a broad concept. Religious physicians and those trained in palliative care experience fewer barriers to providing spiritual care. Additional training in reflections on one’s own perception of spirituality and multidisciplinary training in spiritual care may contribute to the quality of care.
Hamouda et al., 2019, USA [27]	Multicentre cross-sectional descriptive study *n* = 255 (physicians)	To describe the perspectives and practices of American Muslim physicians with respect to R/S discussions, as well as how physician characteristics correlate with them.	Duke University Religiousness Index (DUREL) and Multidimensional Empathy Scale (MES)	More empathetic physicians were reported to be more likely to ask about patients’ R/S, share their own religious ideas and experiences, and encourage patients in their own beliefs and practices. They were also more likely to encourage the discontinuation of unhelpful life-sustaining interventions. They also encouraged their patients to reconcile their own lives. This shows that improving physician empathy may be key to addressing patients’ health-related R/S needs.
Kichenadasse et al., 2017, Australia [25]	Multicentre cross-sectional descriptive study *n* = 69 (physicians)	To explore the current practice, preparedness, and education of Australian oncologists and oncology residents on the provision of spiritual care to their cancer patients.	Spirituality and SpiritualCare Rating Scale (SSCRS)	Most had encountered patients with spiritual care needs during consultations, and less than half perceived that they could meet their spiritual needs. The barriers they identified were a lack of time, a lack of education, and a lack of understanding of spirituality and spiritual care in the health context. A small minority stated that they had received some education on spiritual care, and a few of them stated that the education was adequate. They indicated that they learned how to provide spiritual care on the job or through their own interest and not through specific training.
Koenig et al., 2017, USA [31]	Multicentre cross-sectional descriptive study *n* = 737 (513 physicians and 224 nurses)	To report on the attitudes and practices of health professionals in the largest Protestant health system in the USA (Adventist Health System).	Ad hoc survey	Many stated that a spiritual history should be taken to identify spiritual values, beliefs, and preferences in patients, they were are willing to do so and review the results, although few currently do so. Education, training, and support can help healthcare professionals identify and address patients’ spiritual preferences.
Penderell and Brazil, 2010, Canada [21]	Qualitative study *n* = 6 (physicians)	To seek greater physician understanding of spiritual care in palliative care.	Semi-structured interviews	This study advocated the training and education of palliative physicians in both the spiritual care of patients and the care of their own spirituality.
Seccareccia and Brown, 2009, Canada [32]	Qualitative study *n* = 10 (physicians)	To explore palliative care physicians’ perspectives and experiences of spiritual care and to identify the role of this practice both personally and professionally.	Semi-structured interviews	This study considered the importance of the spiritual dimension in the holistic care of terminally ill persons by physicians and the importance of the spiritual self-care of practitioners.
Smyre et al., 2018, USA [22]	Multicentre cross-sectional descriptive study *n* = 1156 (physicians)	To explore physicians’ beliefs about the relative importance and appropriateness of engaging with patients’ spiritual concerns and physicians’ options for intervention.	Ad hoc survey	Most believe it is essential that patients’ spiritual concerns are addressed at the end of life. The more religious were more likely to believe this and that it is appropriate to always encourage patients to talk to a chaplain. Most stated that, if asked, they would join the family and patient in prayer. Most support a limited role in the provision of spiritual care, although opinions varied according to the religious characteristics of the physicians.

## Data Availability

Not applicable.
